# Low expression of ZHX1 and ZHX2 impacts on the prognosis of chronic lymphocytic leukemia

**DOI:** 10.1186/s40364-021-00263-2

**Published:** 2021-02-04

**Authors:** Natália Ioseph Gladistone Maciel, Luma Dayane Carvalho Filiú-Braga, Francisco Assis Rocha Neves, Eduardo Magalhaes Rego, Antonio Roberto Lucena-Araujo, Felipe Saldanha-Araujo

**Affiliations:** 1 Laboratório de Hematologia e Células-Tronco, Universidade de Brasília, Av. L2 Norte, Brasília, DF 70.910-900 Brazil; 2 Laboratório de Farmacologia Molecular, Universidade de Brasília, Av. L2 Norte, Brasília, DF 70.910-900 Brazil; 3 Laboratório de Hematologia, Universidade de São Paulo, Av. Bandeirantes 3900, Ribeirão Preto, SP 14.048-900 Brazil; 4 Laboratório de Hematologia, Universidade Federal de Pernambuco, Av. Prof. Moraes Rego, Recife, PE 50670-901 Brazil

**Keywords:** ZHX1, ZHX2, Chronic lymphocytic leukemia, Karyotype, Prognosis

## Abstract

Experimental evidence points to the role of Zinc fingers and homeoboxes protein 1 and 2 (ZHX1 and ZHX2) in the development and progression of several types of cancer, including hematological malignancies. Here, we determined whether the altered expression of *ZHX1* and *ZHX2* has clinical implications in patients with CLL. Interestingly, CLL patients with low expression *ZHX1* and *ZHX2* presented higher WBC counts. Importantly, our data showed that CLL patients with cytogenetic alterations presented reduced transcriptional levels of *ZHX1* and *ZHX2* in comparison with patients with normal karyotype. Moreover, when stratifying CLL patients according to the karyotype prognosis value, we observed that the expression of *ZHX1* and *ZHX2* was significantly reduced in CLL patients presenting adverse karyotypes. Finally, we stratified patients according to the number of chromosomal aberrations and observed a negative association between *ZHX1* and *ZHX2* expression and the accumulation of chromosomal abnormalities in CLL patients. Our data showed that the low expression of *ZHX1* and *ZHX2* is associated with a worse prognosis in CLL, followed by a greater number of leukemic cells and unfavorable cytogenetics findings in the diagnosis. Further studies will be important to confirm the prognostic value of ZHX1 and ZHX2 in independent CLL cohorts.

**Supplementary Information:**

The online version contains supplementary material available at 10.1186/s40364-021-00263-2.

To the Editor,

Zinc fingers and homeoboxes protein 1 and 2 (ZHX1 and *ZHX2*) are two members of the zinc-finger and homeobox families, which act as transcriptional repressors. Experimental evidence points to the role of such ZHX members in the development and progression of several types of cancer, including hematological malignancies [1]. Nevertheless, the expression of *ZHX* members on CLL remains unknown.

In this article, we determined whether the altered expression of *ZHX1* and *ZHX2* has clinical implications in patients with CLL. Initially we accessed the BloodSpot database by the International Microarray Innovations in Leukemia (MILE) study group [2], which was performed in a cohort of 73 healthy bone marrow samples and 448 CLL patients. Additionally, *ZHX1* and *ZHX2* expression was determined by quantitative real-time PCR in peripheral blood mononuclear cells (PBMC) obtained from 51 CLL patients, diagnosed according to Matute’s score, and treated at the University Hospital of the Medical School of Ribeirão Preto (University of São Paulo, Brazil). For comparison purposes, 8 samples of peripheral blood B-sorted cells from age-matched healthy volunteers were included. Flow cytometry analysis of ZAP70 expression was performed, following the identification of the CD19+ CD5+ population. Patients presenting at least 20% of leukemic cells expressing ZAP-70 were designated as “ZAP-70 positive”. Cytogenetic analysis was performed by G-banding and according to the results, the prognostic classification of this cohort was determined as favorable, intermediate, and adverse [3, 4]. The statistical analysis is described in the supplementary information file.

Data from the MILE study group showed that *ZHX1* mRNA levels were increased in CLL (*p* ≤ .0001), regardless of the probe analyzed. Using the probe 203556_at, no differences regarding the expression of the *ZHX2* gene between CLL and healthy bone marrow samples were found (*p* > 0.05). Conversely, the probe 1557706_at revealed an increased expression of this transcript in CLL samples (*p* = 0.002). In order to validate these data and explored possible prognostic implications in CLL, we decided to investigate the levels of expression of *ZHX1* and *ZHX2* in 51 patients with CLL and 8 B-cell samples obtained from healthy donors. The median age of patients was 65 years (range: 43–85), and 21 of them were female (41%). Overall, 35 patients were classified as Binet A, followed by 11 Binet B, and 5 Binet C. The expression of the *ZHX1* (*p* > 0.05) and *ZHX2* (*p* > 0.05) genes were similar between CLL and control samples (supplementary Fig. 1). Although we have not explored the functions of ZHX family members at a functional level, we speculate that such contrasting data between in silico analysis and our validation can be explained by the differences between control samples. While the bloodspot database uses bone marrow cells, the primary samples from our court were previously purified B-cell samples.

In order to compare the clinical and laboratorial features of CLL patients grouped according to *ZHX1* and *ZHX2* transcript levels, we adopted the median value of *ZHX1* and *ZHX2* expression as the cut-off level. Clinical and baseline characteristics revealed that patients with low *ZHX1* expression had higher leukocyte (WBC) counts (*p* = 0.03) and more frequent karyotype alterations (*p* = 0.007) indicatives of intermediate and adverse prognosis (*p* = 0.002). Patients with low *ZHX2* expression also showed karyotype changes (*p* = 0.04) categorized into intermediate and adverse risk groups (*p* = 0.09). Furthermore, female patients tended to have lower *ZHX2* expression (p = 0.03) (Table 1). Differential expression of *ZHX1* and *ZHX2* had no impact on Binet stage, platelet number, and expression of ZAP-70 protein (*p* > 0.05). However, when patients with CLL were dichotomized according to the level of expression of *ZHX1* and *ZHX2*, patients with low expression *ZHX1* and *ZHX2* presented higher WBC counts (*p* = 0.002 and *p* = 0.03, respectively). Importantly, our data showed that CLL patients with cytogenetic alterations presented reduced transcriptional levels of *ZHX1* and *ZHX2* in comparison with patients with normal karyotype (*p* = 0.01). Moreover, when stratifying CLL patients according to the karyotype prognosis value (favorable, intermediate, and adverse), we observed that the expression of *ZHX1* and *ZHX2* was significantly reduced in CLL patients presenting adverse karyotypes (*p* = 0.004 and *p* = 0.01, respectively). Finally, we stratified patients according to the number of chromosomal aberrations (0 aberrations: normal karyotype, 1–2 alterations, and ≥ 3 aberrations: complex karyotype) and observed a negative association between *ZHX1* and *ZHX2* expression and the accumulation of chromosomal abnormalities in CLL patients (*p* = 0.002 and *p* = 0.003, respectively) (Fig. 1).

**Table 1:** Clinical and Baseline characteristics

Characteristics	All Patients (%)	ZHX 1		***p***	ZHX2		***p***
		Low	High		Low	High	
**Age, y** Median (range)	65 (43, 85)	65.5 (54, 80)	59 (43, 85)	.44	66.5 (54, 80)	59 (43, 85)	.15
**Sex**				.24			**.01**
Female	21 (41.1)	9 (34.6)	12 (48)		5 (20.8)	13 (56.5)	
Male	30 (58.9)	17 (65.4)	13 (52)		19 (79.2)	10 (43.5)	
**Binet**				.26			.60
A	35 (68.7)	17 (68)	18 (69.2)		16 (48.5)	17 (51.5)	
B	11 (21.5)	4 (16)	7 (27)		5 (50)	5 (50)	
C	5 (9.8)	4 (16)	1 (3.8)		3 (75)	1 (25)	
**Platelets**				.16			.55
median (range), X10^9^ /L	142.5 (11, 312)	137.5 (15, 261)	147 (11, 312)		141.5 (15, 312)	140 (11, 284)	
**WBC**				**.03**			.15
median (range), X10^9^ /L	45.2 (7.8, 170.1)	61.1 (29, 170.1)	23.4 (7.8, 93.2)		45.3 (10.5, 170.1)	30.1 (7.8, 93.2)	
**ZAP-70**^a^				.18			.37
Negative	16 (32)	6 (24)	10 (40)		7 (30.4)	9 (39.1)	
Positive	34 (68)	19 (76)	15 (60)		16 (69.6)	14 (60.9)	
**Karyotype**				**.007**			**.04**
Normal	12 (23.5)	2 (7.7)	10 (40)		3 (12.5)	9 (39.1)	
Abnormal	39 (76.5)	24 (92.3)	15 (50)		21 (87.5)	14 (60.1)	
**Cytogenetic risk**				**.002**			**.09**
Favorable	14 (27.5)	2 (7.7)	12 (48)		4 (16.7)	10 (43.5)	
Intermediate	20 (39.2)	11 (42.3)	9 (36)		11 (45.8)	9 (39.1)	
Adverse	17 (33.3)	13 (50)	4 (16)		9 (37.5)	4 (17.4)	
**Number of karyotypic changes**				**.003**			**.01**
0	12	2 (7.7)	10 (40)		3 (12.5)	9 (39.1)	
1 or 2	26	13 (50)	13 (52)		13 (54.2)	13 (56.5)	
3 or more	13	11 (42.3)	2 (8)		8 (33.3)	1 (4.4)	

Values represent number (percentage), or median (range) when indicated in the row headings

^a^ Missing values were excluded for statistical analysis

Statistically significant differences are in bold

**Fig. 1:**
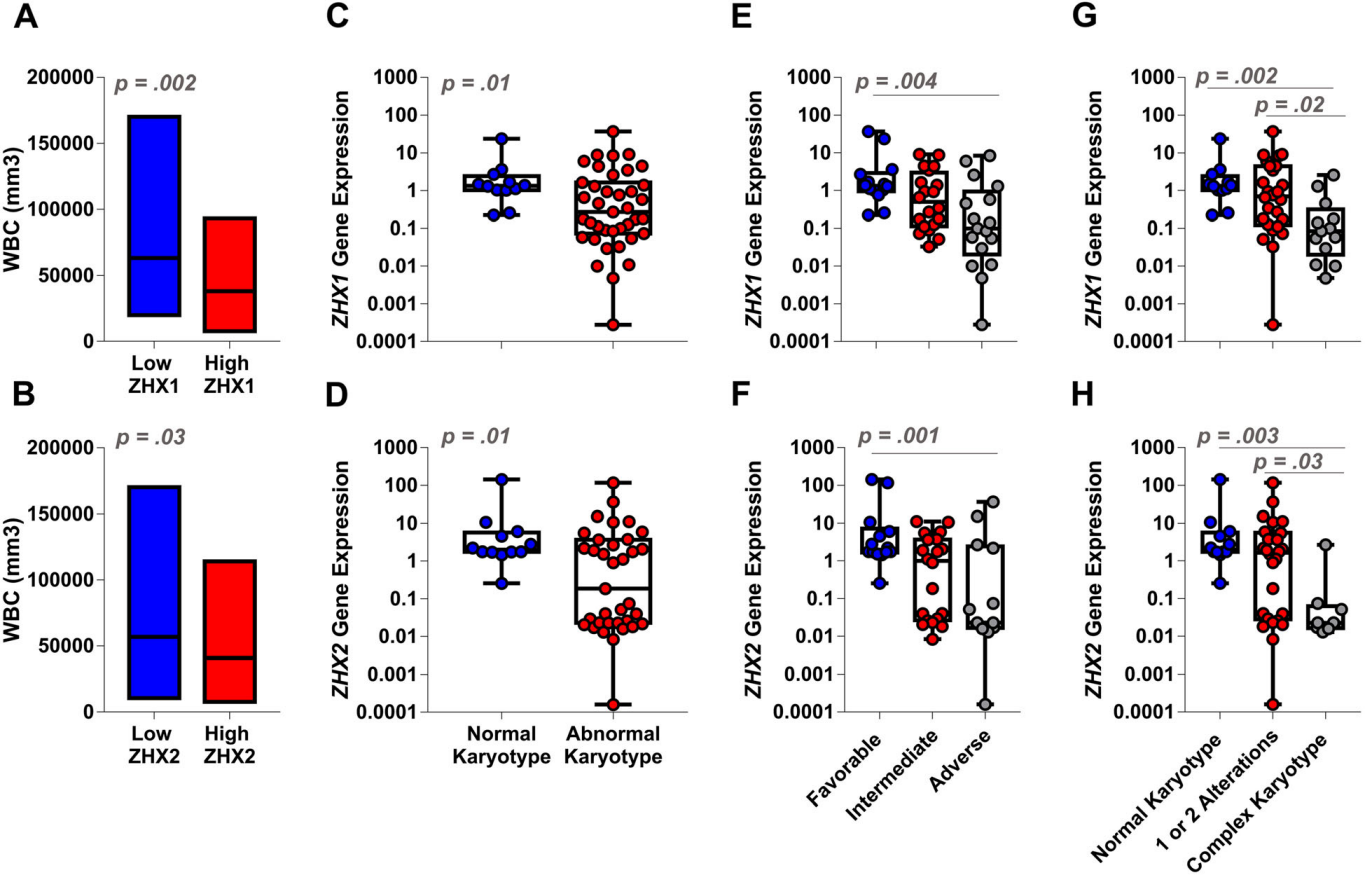
*ZHX1* and *ZHX2* expression in CLL and association with WBC and cytogenetic findings. CLL patients with low expression of *ZHX1* (**a**) and *ZHX2* (**b**) showed higher WBC counts. Low expression of *ZHX1* and *ZHX2* was significantly associated with abnormal (**c**, **d**), adverse (**e**, **f**), and complex (**g**, **h**) karyotype. Mann–Whitney test was employed to compare differences between two groups. Kruskal–Wallis followed by the multiple comparison test (Dunn’s) was used to compare differences between three groups

Our data showed that the low expression of *ZHX1* and *ZHX2* is associated with a worse prognosis in CLL, followed by a greater number of leukemic cells and unfavorable cytogenetics findings in the diagnosis [5]. Recently, several studies have been conducted to unravel the influence of members of the ZHX family on cancer. Interestingly, *ZHX1* was found to be down-regulated in renal cell carcinoma, and the mRNA reduction of this gene was associated with lower survival rates [6]. *ZHX1* is also downregulated in gastric cancer, and the reduced expression of this gene has been associated with other clinical parameters that are indicative of aggressive disease [7]. *ZXH2* was found to have reduced expression in hepatocellular carcinoma, and the decreased levels of this gene were associated with the low survival of patients [8]. Moreover, the impact of ZHX2 on hematological neoplasms has been investigated. It was demonstrated that patients with multiple myeloma who have reduced *ZHX2* expression tend to have a worse prognosis, with resistance to chemotherapy and unfavorable karyotype [9]. Besides, this gene acts as a tumor suppressor in Hodgkin lymphoma [10]. Considering our findings, we hypothesize that ZHX1 and ZHX2 may also play a role as tumor suppressor in CLL, the aberrant expression of these genes possibly contributing to an increase in the number of malignant cells and chromosomal instability.

To the best of our knowledge, our study represents the first step to demonstrate the prognostic impact of ZHX members in CLL. However, it is important to highlight that in our cohort, only 14% of the patients in the favorable prognosis group have del (13q), while in the adverse prognosis group, del (17p) and 11q23 is found in 29% of cases. We cannot rule out that some classic chromosomal changes may have been underestimated in this study. The fact that the samples were not analyzed using FISH method to identify these chromosomal alterations represents a limitation of this work. Further studies will be important to characterize the functional role of ZHX1 and ZHX2 in this leukemia and also to confirm the prognostic value of both genes in independent CLL cohorts.

## Supplementary Information

**Additional file 1: Fig. S1**. Gene expression of *ZHX1* and *ZHX2* in CLL. *ZHX1* gene expression was analyzed in CLL patients by accessing the probes 223213_s_at (A) and 223214_s_at (B) available in the Blood Spot database. (C) *ZHX1* gene expression also was determined in CLL samples by real-time quantitative PCR. *ZHX2* gene expression was analyzed in CLL patients, by accessing the probes 203556_at (D) and 1557706_at (E) available in the Blood Spot database. (F) *ZHX2* gene expression also was determined in CLL samples by real-time quantitative PCR. Horizontal bars represent the median expression of the genes. Mann–Whitney test was employed to compare differences between the groups.

**Additional file 2.**

## Data Availability

All data underlying the findings are fully available.

## Abbreviations

CLL: Chronic lymphocytic leukemia
MILE: Microarray innovations in leukemia
PBMC: peripheral blood mononuclear cells
ZHX 1: Zinc fingers and homeoboxes protein 1
ZHX2: Zinc fingers and homeoboxes protein 2
