# Supplementary material for: Low expression of ZHX1 and ZHX2 impacts on the prognosis of chronic lymphocytic leukemia
Source: Biomark Res. 2021 Feb 4;9:10. doi: 10.1186/s40364-021-00263-2 (PMC7863484; doi:10.1186/s40364-021-00263-2)
Supplement: Supplementary file 2 — Additional file 2. [file 40364_2021_263_MOESM2_ESM.docx]

**Statistical analyses**

The Mann–Whitney test was used for numerical comparisons between two groups. The influence of ZHX1 and ZHX2 expression on cytogenetic findings was determined by Kruskal–Wallis followed by the multiple comparison test (Dunn’s). Fisher’s exact test or Chi-square test, as appropriate, were used to compare categorical variables. Using survival receiver operating characteristic (ROC) curve analysis [1] and the C index [2] we dichotomized patients into two groups according to the median value of ZHX1 (low expression, < 0.65; high expression, ≥ 0.65) and ZHX2 expression (low expression, < 1.72; high expression, ≥ 1.72). Statistical analyses were performed using Prism 7 Software, and the threshold for significance was p < 0.05.

[1] Heagerty PJ, Zheng Y. Survival model predictive accuracy and ROC curves. Biometrics. 2005;61:92–105.

[2] Harrell FE Jr, Califf RM, Pryor DB, Lee KL, Rosati RA. Evaluating the yield of medical tests. JAMA. 1982;247:2543–6.
